# Comparative Analysis of Metabolite Changes in Huangjiu During Different Aging Periods Using HRMS Metabolomics

**DOI:** 10.3390/metabo15050298

**Published:** 2025-04-30

**Authors:** Yue E, Zhuang Wang, Hongbin Guo

**Affiliations:** 1 School of Chemical Engineering and Technology, Tianjin University, Tianjin 300072, China; z_wang@tju.edu.cn; 2 Zhejiang Institute of Tianjin University, Shaoxing 312300, China

**Keywords:** Huangjiu, high-resolution mass spectrometry, metabolomics, characteristic metabolites of Huangjiu aging

## Abstract

Background: Huangjiu, a traditional Chinese fermented alcoholic beverage, exhibits a multifaceted chemical profile comprising diverse metabolites, such as lipids, amino acids, and phenolic compounds. The age of the wine is an important indicator of its quality and is a primary reference for purchasing decisions. Methods: This study employs high-resolution mass spectrometry to perform metabolomics analysis on Huangjiu of varying ages and uses multivariate statistical analysis to characterize the chemical features of different types of Huangjiu. This research investigates the Huangjiu aged from 3 to 30 years, involving samples of five different aging periods. Results: A total of 415 compounds were detected across all samples, including 147 differential metabolites. It was observed that, as the aging of Huangjiu increased, the relative content of most metabolites showed a rising trend. However, 19 metabolites, mainly lipids and lipid-like molecules, decreased in concentration over time. This finding highlights significant differences in metabolite composition among Huangjiu of different ages. Furthermore, 19 characteristic differential metabolites were predicted as markers for distinguishing Huangjiu of different ages. Conclusions: This study provides theoretical and material foundations for quality control, health benefits, and industrial development of Huangjiu.

## 1. Introduction

Huangjiu, also known as Chinese rice wine, is a traditional alcoholic beverage made from glutinous rice, millet, and japonica rice. Huangjiu, together with wine and beer, is collectively known as the three ancient liquors of the world [1]. The history of Huangjiu can be traced back several thousand years, making it an important component of traditional Chinese culture. It is not merely a beverage but also carries a rich historical and cultural significance. Huangjiu is known for its rich aroma and mellow flavor [2]. Its production process generates bioactive compounds, such as polyphenols, amino acids, and organic acids, which have been studied for their biochemical properties [3,4,5,6,7].

Fermentation is an ancient, natural, and economical method of food processing and preservation. Despite its use and development over centuries, our understanding of the basic fermentation process remains limited. With the advent of analytical tools like omics technologies, we are striving to build more knowledge about fermented products. By studying fermented foods through metabolomics, we can predict the taste and nutritional quality of fermented products and observe their metabolic changes [8,9]. Park et al. [10] utilized metabolomics technology to analyze the metabolic changes during the fermentation process of kimchi, revealing the formation mechanisms of its flavor and nutritional quality. Lee et al. [11] predicted the flavor and nutritional quality of fermented soybean products using metabolomics technology and analyzed their metabolite composition. Zhang et al. [12] employed metabolomics technology to analyze the metabolic pathways during cheese fermentation, revealing the mechanisms behind its flavor formation. Wang et al. [13] analyzed the flavor and health benefits of fermented tea using metabolomics technology, revealing changes in its metabolite composition.

In recent years, metabolomics technology has also been widely applied in the study of alcoholic beverages, investigating the changes in metabolites during fermentation and their impact on flavor and quality [14,15,16]. Some volatile organic compounds in Huangjiu can affect the aroma of the wine and are an important component of the overall flavor profile. The volatile components of Huangjiu are highly complex, comprising esters, alcohols, acids, carbonyl compounds, and other aromatic compounds, making Huangjiu’s aroma a composite scent. The primary volatile organic compounds in Huangjiu include monosaccharides, acids, sulfides, esters, and aldehydes [17]. Using comprehensive two-dimensional gas chromatography-time of flight mass spectrometry (GC×GC-TOFMS) technology, Peng et al. [18] compared traditional handmade and mechanized production of Huangjiu using GC-MS metabolomics, finding significant differences in ester content during the post-fermentation stage between the two production methods to explain why mechanized Huangjiu lacks the flavor profile of handmade Huangjiu. Wang et al. [19] identified 106 aroma-active substances through GC-O/AEDA and odor value estimation (Osme) from compounds separated by solvent-assisted flavor evaporation (SAFE) distillation and quantitatively analyzed 47 of them. Some non-volatile compounds, while influencing taste and mouthfeel, can also alter the aroma characteristics of Huangjiu. Additionally, these compounds have significant health implications, including peptides, polyphenols, alkaloids, terpenes, amino acids, and organic acids [20]. A non-targeted metabolomics analysis of Huangjiu from three different regions (Shaoxing, Jimo, and Fangxian) was conducted using ultra-performance liquid chromatography-electrospray ionization-triple quadrupole linear ion trap-MS/MS (UPLC-ESI-Q trap-MS/MS), identifying a total of 1146 metabolites, 997 of which were identified for the first time [21]. Fu et al. [22] identified over 500 polyphenols in Huangjiu, including 45 potential bioactive peptides and 3 sensory active peptides, using UPLC-ESI-MS/MS. The study found that most of these bioactive peptides are dipeptides and tripeptides with ACE (angiotensin-converting enzyme) bioactivity, which can significantly lower blood pressure in the renin-angiotensin system.

Huangjiu is rich in various beneficial components, such as polyphenols, organic acids, and vitamins. The flavor components of Huangjiu vary depending on its raw materials, fermentation agents, sugar content, and production processes [23]. Moreover, the flavor of Huangjiu can change due to regional and environmental factors. Another important factor is the age of wine. The age of Huangjiu is an important indicator of its quality. Generally, the longer the aging period, the better the flavor, aroma, and taste of the Huangjiu. The changes in aging are closely related to the chemical composition of Huangjiu and its storage conditions, while also carrying profound cultural significance. Through scientific evaluation and proper storage, the aging potential of Huangjiu can be fully realized, enhancing its quality and value. However, research on the differences in metabolites of Huangjiu from different vintages is still not detailed enough. Given that Huangjiu undergoes a complex fermentation process that is either open or semi-open, its composition is challenging to analyze. Therefore, it is necessary to strengthen research on the metabolites in Huangjiu from different years using metabolomics and multivariate statistical analysis. A more detailed analysis of the metabolites in Huangjiu would lead to a better understanding of its flavor characteristics and enrich the research on Huangjiu’s flavor, which is essential for deeper research, understanding, and promotion of Huangjiu. It is also important to adopt modern scientific techniques to ensure strict and unified evaluation standards for Huangjiu during production, storage, and distribution. This will further enhance the fairness and accuracy of wine evaluations.

Therefore, this study employs metabolomics and multivariate statistical analysis to systematically characterize metabolite changes in Huangjiu across different aging periods. The findings aim to establish a theoretical foundation for quality control, health benefits, and industrial standardization of Huangjiu.

## 2. Materials and Methods

### 2.1. Experimental Materials

Chromatography-grade (>99.99%) acetonitrile, methanol, and formic acid were purchased from Thermo Fisher Scientific (Waltham, MA, USA). Ultrapure water was prepared using a Millipore-Q water purification system (Millipore Corporation, Burlington, MA, USA).

The Huangjiu samples used in this study are commercially available. All samples were stored in a refrigerator at 4 °C prior to analysis. Specific information about the Huangjiu samples is as follows: Three-Year-Aged Shaoxing Huadiao, Five-Year-Aged Shaoxing Huadiao, Ten-Year-Aged Shaoxing Huadiao, Twenty-Year-Aged Shaoxing Huadiao, and Thirty-Year-Aged Shaoxing Huadiao. The total number of analyzed samples is 30, with 6 samples per age group (year) across 5 years.

### 2.2. Analysis of Metabolites

#### 2.2.1. UPLC Conditions

Waters quadrupole time-of-flight tandem liquid chromatography-mass spectrometry system (AQUITY Premier/SYNAPT XS, Waters Corporation, Milford, MA, USA). Liquid Chromatography Conditions: Column: ACQUITY Premier BEH C18 Column, 1.7 μm, 2.1 × 50 mm. Mobile Phases: Solvent A (0.1% formic acid in water) and Solvent B (0.1% formic acid in acetonitrile). Gradient Elution Program: Start with 2% B; linearly increase B to 98% over 10 min; hold at 95% for 3 min; decrease B back to 2% between 13.00–13.10 min, and equilibrate at 2% until 15 min. Flow Rate: 0.4 mL/min, Column Temperature: 45 °C, Injection Volume: 10 μL.

#### 2.2.2. Mass Spectrometry Conditions

High-resolution mass spectrometry was used in Resolution mode for primary data acquisition. Electrospray Ionization (ESI) Source Parameters: Capillary voltage: +3000 V in positive ion mode and −2000 V in negative ion mode. Desolvation temperature: 500 °C, cone voltage: 40 V, source temperature: 120 °C. Data were acquired in MSe Continuum mode, which enables simultaneous acquisition of both precursor (MS1) and fragment (MS2) ion data across the full scan range of m/z 50–1200; ramp transfer collision energy was 10–60 V.

#### 2.2.3. Data Processing and Statistical Analysis

Data Acquisition and Processing: Initial data were acquired using Mass Lynx V4.2 and processed with Progenesis QI software (version 3.0) for peak picking, alignment, and sample grouping. Raw LC-MS features were annotated using HMDB (Human Metabolome Database), KEGG (Kyoto Encyclopedia of Genes and Genomes), and ChEBI (Chemical Entities of Biological Interest). Annotation confidence was assigned according to Schymanski et al. [24].

Unsupervised PCA (principal component analysis) was performed using the statistics function prcomp within R (www.r-project.org, accessed on 17 May 2024). The data were unit variance scaled before unsupervised PCA. Metabolite abundance data were normalized using unit variance scaling (UV), and heatmaps were generated via the ComplexHeatmap package in R software (version 4.2.0) to visualize hierarchical cluster analysis (HCA) of metabolite accumulation patterns across different samples. For OPLS-DA, the raw data underwent log2 transformation followed by mean-centering. The analysis was performed using the OPLSR.Anal function from the MetaboAnalystR package in R.

Further analysis of the identified metabolites was conducted using the online analysis platform, defining differential metabolites by VIP (Variable Importance in Projection) > 1, *p* value < 0.05, and Fold Change (FC) ≥ 2 or ≤0.5. The identified metabolites were then subjected to Principal Component Analysis (PCA), Orthogonal Partial Least Squares-Discriminant Analysis (OPLS-DA), and Cluster Analysis.

## 3. Results and Discussion

### 3.1. Untargeted Metabolomic Profiling

A total of 415 metabolites were detected and categorized into the following classes: Lipids and lipid-like molecules (125): including glycerophospholipids, fatty acyls, and sterol lipids. Amino acids, peptides, and analogs (90): including proteinogenic amino acids, dipeptides, and modified amino acids. Flavonoids (2): identified as flavones and flavonol glycosides. Phenolic acids (3): subclassified into hydroxybenzoic acids and hydroxycinnamic acids. Organic heterocyclic compounds (55): including pyridines, indoles, and purines. Benzenoids (25): aromatic compounds such as benzene derivatives and polyphenols. Organic oxides (15): including epoxides and cyclic ethers. Phenylpropanoids and polyketides (11): subclasses include lignans and polyketide antibiotics. Organic nitrogen compounds (9): including amines and guanidines. Organic acids (8): carboxylic acids and short-chain fatty acids. Alkaloids and derivatives (7): examples include isoquinoline alkaloids and tropane derivatives. Nucleosides, nucleotides, and analogs (5): including purine nucleosides and pyrimidine nucleotides. Coumarins and derivatives (4): identified as simple coumarins and furanocoumarins. Others (56): minor classes such as carbohydrates, vitamins, and cofactors.

**Figure 1:**
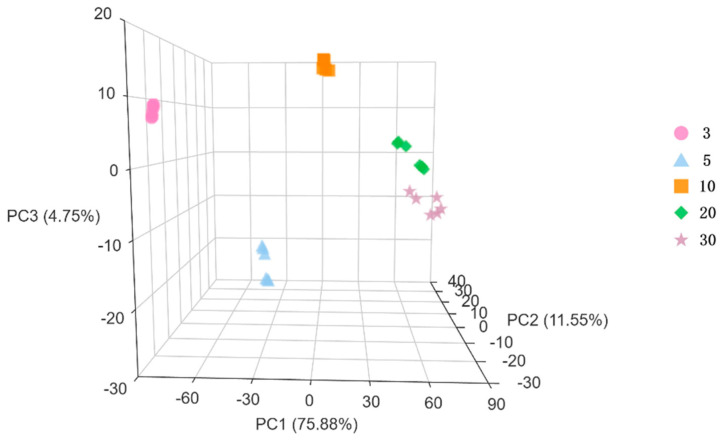
PCA analysis of differential metabolites in Huangjiu samples of five different ages.

When PCA was applied to the Huangjiu samples, the results (see Figure 1) showed a clear separation trend among the 3-year, 5-year, and 10-year samples, indicating significant differences between these samples. However, the 20-year and 30-year samples showed some overlap, suggesting a certain degree of similarity between these two groups. The parallel samples of Huangjiu from the same aging period overlapped closely, indicating high reproducibility between the samples. The PCA results can effectively reflect the differences in metabolites between Huangjiu of different ages. This suggests that the aging process plays a significant role in influencing the characteristics of Huangjiu.

To more clearly illustrate the overall metabolic differences, OPLS-DA analysis was performed on the samples. OPLS-DA is applied to identify differential metabolites between different groups. Through OPLS-DA analysis, a VIP score is calculated for each metabolite, where a higher VIP score indicates that the metabolite plays a more significant role in distinguishing between the two sample groups. The R^2^Y value represents the interpretability of the model; the closer R^2^Y is to 1, the more information the model can explain regarding the classification of the two groups, and the greater the differences between the groups. The two dashed lines represent the regression lines for R^2^Y and Q^2^.

The results (see Figure S1a) showed that Huangjiu samples aged for 3 years were primarily distributed on the positive half-axis of PC1 and PC2, 5-year samples were mainly on the positive half-axis of PC1 and PC2, 10-year samples were mainly on the negative half-axis of PC1 and positive half-axis of PC2, 20-year samples were mainly on the negative half-axis of PC1 and PC2, and 30-year samples were primarily distributed on the positive half-axis of PC1 and the negative half-axis of C2. To prevent data overfitting and ensure the model’s validity, a permutation test was performed to obtain a random model (see Figure S2a), resulting in R^2^Y = 0.991 and Q^2^ = 0.979, both of which are close to 1, indicating that the model is stable and reliable.

**Figure 2:**
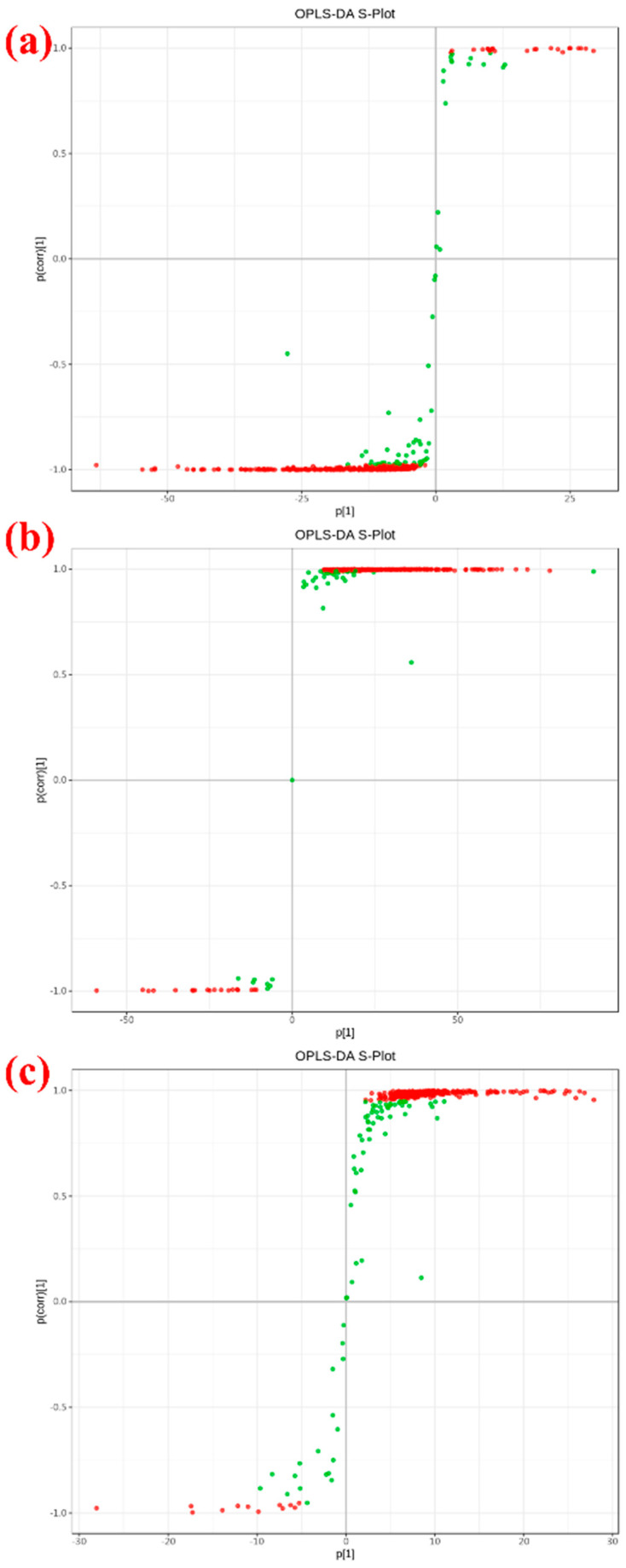
OPLS-DA S-plots of different group comparison (**a**) 3-year vs. 10-year, (**b**) 3-year vs. 30-year, (**c**) 10-year vs. 30-year.

To better demonstrate the impact of aging on the differences in Huangjiu metabolites, the Huangjiu samples aged 3 years, 10 years, and 30 years were compared individually. Figure 2 shows the OPLS-DA plots for the 3-year vs. 10-year, 3-year vs. 30-year, and 10-year vs. 30-year comparisons, respectively. As seen, the comparison models between 3 years and 10 years (see Figure S1b), 3 years and 30 years (see Figure S1c), and 10 years and 30 years (see Figure S1d), both of R^2^Y and Q^2^ are close to 1, the models are relatively stable. Additionally, the three groups show no overlap in their respective OPLS-DA plots, indicating high reliability. Differential metabolites can be identified through VIP analysis.

The S-plot diagrams compare the specific groups mentioned above (3 years vs. 10 years, 3 years vs. 30 years, and 10 years vs. 30 years). Metabolites that are located far from the origin of the coordinates significantly contribute to the differences between the samples. The metabolites near the top-right and bottom-left corners of the plots indicate more pronounced differences. In Figure 2, the red dots represent VIP > 1, while the green dots represent VIP < 1. A total of 353 metabolites (Figure 2a, 3 years vs. 10 years), 367 metabolites (Figure 2b, 3 years vs. 30 years), and 340 metabolites (Figure 2c, 10 years vs. 30 years) were found to have VIP values greater than 1. These metabolites are the furthest from the origin and appear in both the positive and negative directions on the S-plot diagrams. Therefore, these metabolites can be considered key metabolites for distinguishing differences in Huangjiu.

**Figure 3:**
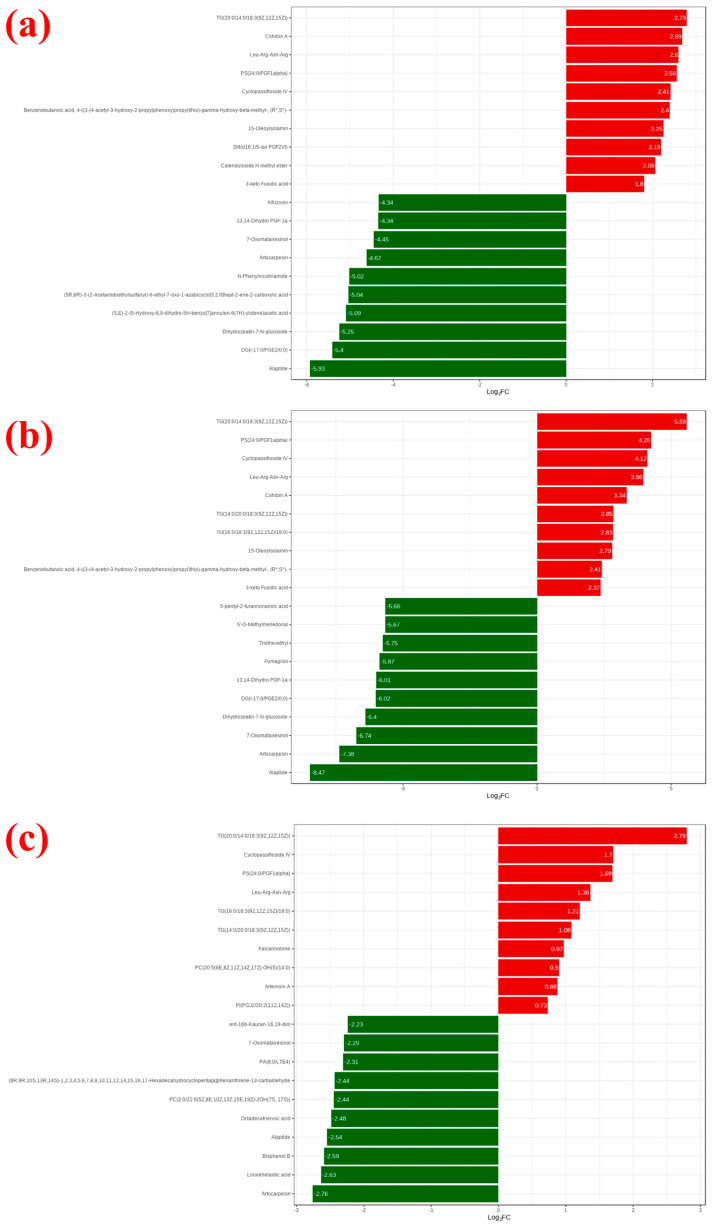
Top 20 metabolites with the highest fold changes in each group comparison; red indicates up-regulated metabolites, while green indicates down-regulated metabolites (**a**) 3-year vs. 10-year, (**b**) 3-year vs. 30-year, (**c**) 10-year vs. 30-year.

Based on the FC values of the metabolites in the comparison groups, a clearer understanding of the overall metabolic changes among different samples can be obtained. Metabolites that meet the criteria FC ≥ 2 or FC ≤ 0.5 were selected, resulting in 203 metabolites for 3 years vs. 10 years, 203 metabolites for 3 years vs. 30 years, and 98 metabolites for 10 years vs. 30 years. Figure 3 shows the top 10 upregulated and downregulated metabolites. In Figure 3, red represents upregulated metabolites (i.e., in the experimental group, these metabolites are positively regulated and their expression levels increase compared to the control group), while green represents downregulated metabolites (i.e., in the experimental group, these metabolites are suppressed and their expression levels decrease compared to the control group). For example, compared to 10 years and 30 years, the FC value of Alaptide in 3 years is lower, showing downregulation. Conversely, TAG(52:3) in 3 years has the highest FC value and is upregulated. Compared to 30 years, N-lauroyl histidine in 10 years has a higher FC value and is also upregulated. Table S1 presents the metabolites that exhibited up-regulation (down-regulation) across all three groups. Metabolomic analysis revealed consistent upregulation of the following metabolites across all three experimental groups: Cyclopassifloside IV, PS(24:0/PGF1α), Leu-Arg-Asn-Arg, TG(20:0/14:0/18:3(9Z,12Z,15Z)), and Falcarinolone. Conversely, significant downregulation was observed in alpha-eleostearic acid, 7-oxomatairesinol, artocarpesin, and PC(2:0/22:6(5Z,8E,10Z,13Z,15E,19Z)-2OH(7S,17S)).

**Figure 4:**
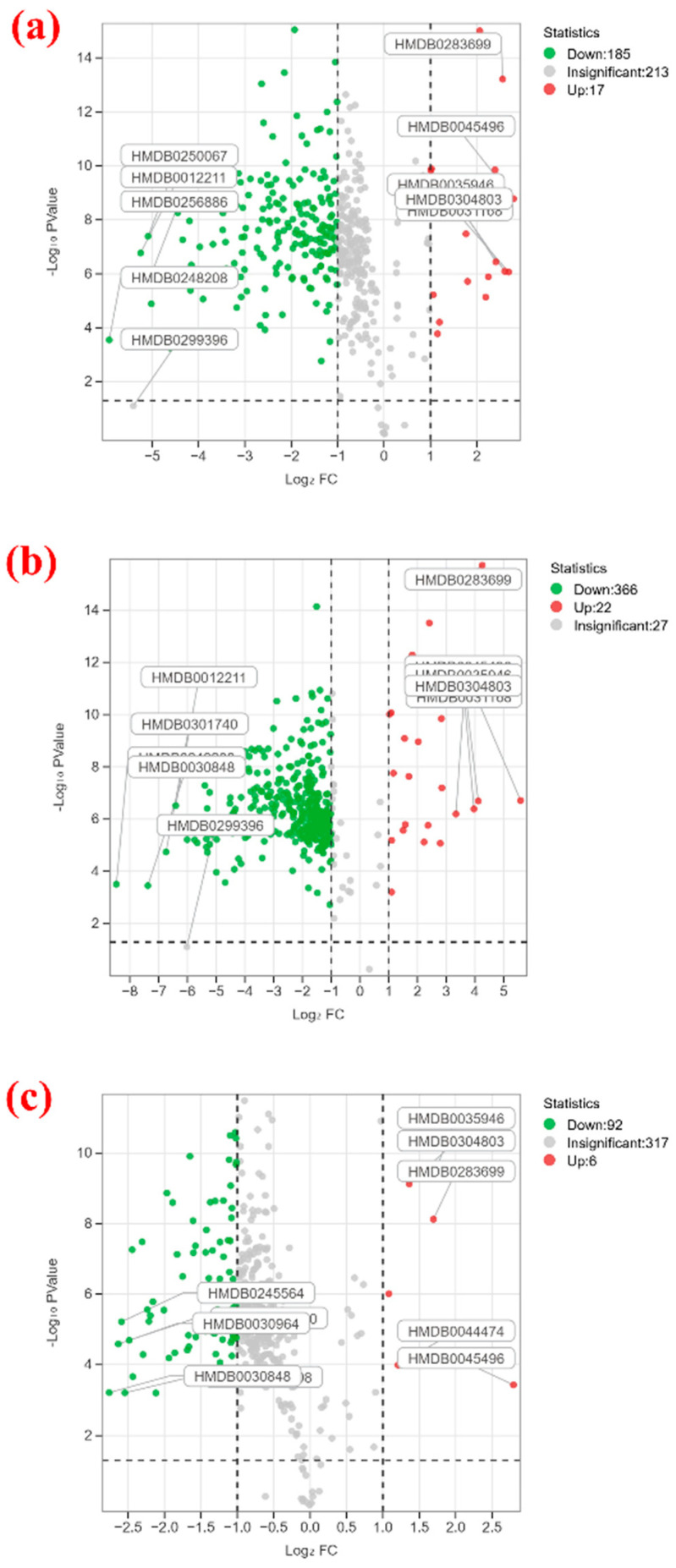
Volcano plots of the differential metabolites of different group comparisons; (**a**) 3-year vs. 10-year, (**b**) 3-year vs. 30-year, (**c**) 10-year vs. 30-year.

Volcano plots combine statistical significance (*p* value) and fold change (FC value) to quickly and intuitively identify parts of the data with significant changes. To better understand the differences in metabolites between 3 years, 10 years, and 30 years, volcano plots (see Figure 4) were created with *p* ≤ 0.05, FC ≤ 0.5, or FC ≥ 2 as criteria. In these plots, red and green dots represent upregulated and downregulated metabolites, respectively, while gray dots represent non-differential metabolites.

From the plots, it is observed that compared to 10 years, there are 185 downregulated and 17 upregulated metabolites in 3 years (Figure 4a); in the comparison between 3 years and 30 years (Figure 4b), there are 22 upregulated and 366 downregulated metabolites; compared to 30 years, there are only 6 upregulated and 92 downregulated metabolites in 10 years (Figure 4c). Notable metabolites include PS (24:0/PGF1alpha), TAG(52:3), Cyclopassifloside IV, 3-[(17Z)-13,14-dihydroxy-triene-17-yl]-5-methyl-5H-furan-2-one, and aspartyl-alanylasparagine, which are upregulated in comparisons of 3 years vs. 10 years and 30 years. DG(i-17:0/PGE2/0:0), alpha-stearic acid, and dihydroxycornan-7-N-glucoside are downregulated in both 3 years vs. 10 years and 30 years.

**Figure 5:**
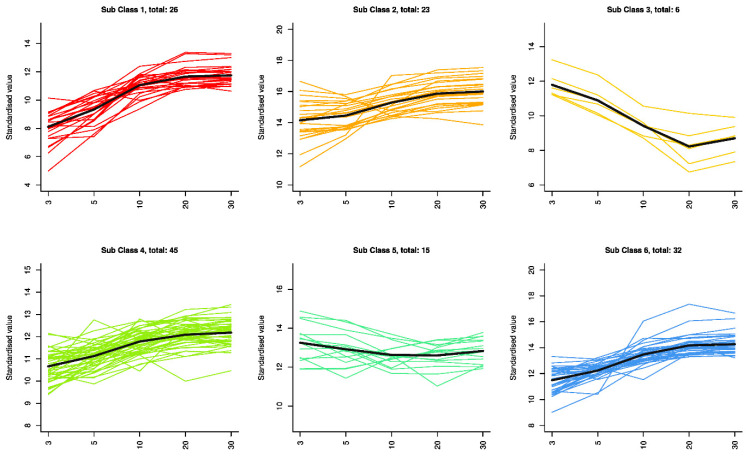
K-means clustering groups of the differential metabolites. Y axis: the standardized content of per metabolite; X axis: different groups.

Using the K-means method, differential metabolites were divided into six subclasses. As shown in Figure 5, each group contains 26, 23, 6, 45, 15, and 32 metabolites, respectively. In subclasses 1, 2, 4, and 6, the relative content of metabolites in the 3-year, 5-year, 10-year, and 20-year samples gradually increased, while the relative content tended to stabilize in the 20-year and 30-year samples. In subclass 2, the highest number of amino acid metabolites is observed, totaling nine types, with the relative content of all except 15-octadecenoyl sorlamine gradually increasing. In subclass 3, six metabolites (Cohibin A, 3-keto-fusidic acid, L-prolylproline, PS (24:0/PGF1alpha), SM(d16:1/5-iso PGF2VI), and Leu-Arg-Asn-Arg) differ from those in other subclasses, primarily showing a decrease in relative content with increasing years, starting to increase after 20 years. The relative content of the 15 metabolites in subclass 5 tends to stabilize, showing little overall variation, such as (S)-Multifidol 2-[apiosyl-(1- > 6)-glucoside] and Calenduloside H methyl ester.

### 3.2. Analysis of Differential Metabolites

Based on the OPLS-DA analysis results, differential metabolites were screened using the criteria of VIP > 1, *p* < 0.05, and FC ≥ 2 or ≤0.5. A total of 147 differential metabolites were detected, accounting for 35.42% of all detected metabolites. These differential metabolites may be related to the formation and variation of the sensory characteristics of Huangjiu, such as taste and aroma. Among the 147 differential metabolites identified, there are 40 lipids and lipid-like molecules, 31 amino acids and their derivatives, 24 organic heterocyclic compounds, 7 phenylpropanoids and polyketides, 5 aromatic compounds, 5 organic oxidants, 3 nucleosides, nucleotides, and analogs, 3 organic nitrogen compounds, 2 organic acids and derivatives, 2 alkaloids and their derivatives, 1 coumarin and its derivatives, and 24 others.

The flavor of Huangjiu depends on the types and relative amounts of amino acids, which play a significant role in its taste [25]. Among the 31 amino acids detected, most have the highest relative abundance in older vintages. However, some amino acids do not follow this trend. For instance, Leu-Arg-Asn-Arg is most abundant in the 3-year samples but has the lowest relative abundance in the 20-year and 30-year samples. Lysine, valine, and isoleucine show the lowest relative abundance in 5-year samples, following a trend of initial decrease and later increase, similar to previous findings [26]. This trend might be due to protein conversion into amino acids, leading to an overall increase in their relative content. In summary, the flavor of Huangjiu improves with aging, but the rate of improvement can be affected by changes in certain amino acids. Such trends align with studies demonstrating that amino acid profiles in fermented foods are shaped by both enzymatic processes and environmental factors [27], underscoring the need for targeted analyses of peptide stability in aging Huangjiu.

**Figure 6:**
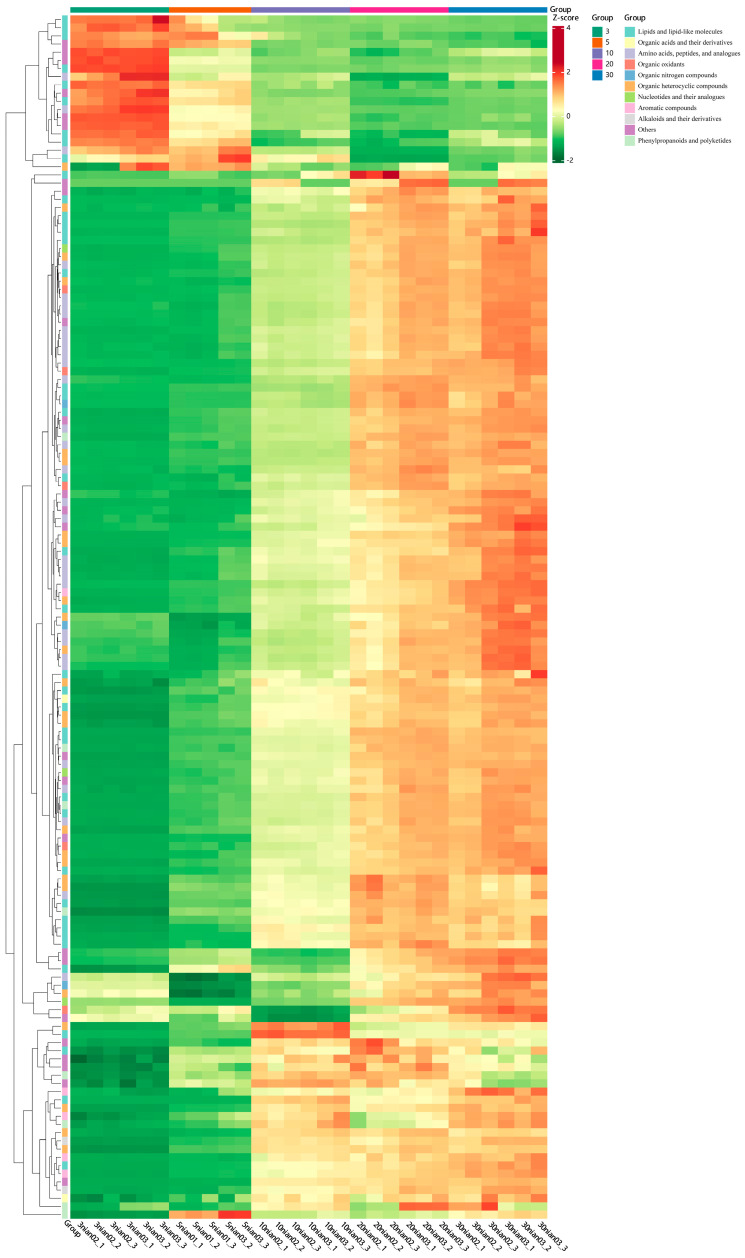
HCA analyses of differential metabolites in Huangjiu samples of five different ages.

Lipids and lipid-like molecules are one of the main components contributing to the aroma of Huangjiu [28]. Among the 40 lipid compounds detected, most show an increasing trend in relative abundance with aging, although a few exhibit the opposite trend. For example, 3-keto-fusidic acid, Cohibin A, 15-Oleoylsolamin, Artemoin A, and Calenduloside H methyl ester have the highest relative abundance in 3-year and 5-year samples, while (4E,7E,10E,13E)-hexadeca-4,7,10,13-tetraenoic acid, 13,14-dihydro PGF-1a, and 5-pentyl-2-furannonanoic acid have the highest abundance in 30-year samples, as shown in Figure 6. Glycerophosphocholine and DG (22:6(4Z, 7Z, 10Z, 13Z, 16Z, 19Z)/22:5(7Z, 10Z, 13Z, 16Z, 19Z)/0:0) may increase in content as phosphoethanolamine undergoes glycerophospholipid metabolism or as 1-alkenylglycerophosphocholine undergoes ether lipid metabolism. These findings corroborate the hypothesis that prolonged aging promotes the stabilization of lipid-derived volatiles, a phenomenon also documented in rice wine aging studies [25].

Coumarin, also known as o-hydroxycinnamic acid lactone, is a class of natural products with a benzopyrone core. It exhibits a wide range of biological and pharmacological activities, such as anti-inflammatory, anti-tumor, and anti-HIV properties [29]. Among the detected compounds, 7-[(6-hydroxy-3,7-dimethyl-2,7-octadienyl)oxy]-2H-1-benzopyran-2-one showed the highest relative content in 30-year-aged Huangjiu.

Phenylpropanoids are a class of naturally occurring compounds characterized by a benzene ring linked to a three-carbon chain (C6-C3 unit). They possess various biological activities, including anti-tumor, anti-inflammatory, insecticidal, antiplatelet aggregation, and antiviral effects [30,31]. For example, osmanthuside A, detected as a flavoring agent with sweet, floral, and fruity notes, showed an increasing relative content over the years in the heatmap analysis. This may be one of the contributing factors to the enhanced aroma of Huangjiu with prolonged aging.

Organic acids in Huangjiu not only influence its acidity but also profoundly affect the acid-base balance of the wine and the formation of ester compounds [32]. The detection results revealed that the relative content of anserine increased steadily over time. This may contribute to subtle changes in the flavor profile of Huangjiu.

### 3.3. Prediction of Characteristic Metabolites of Huangjiu Aging

Hierarchical Clustering Analysis (HCA) is a process of grouping data objects into multiple classes consisting of similar objects. The expression values of metabolites that are higher than the average are marked in red, while those lower than the average are marked in green. The intensity of the color reflects the degree of difference from the average. In Figure 6, the horizontal axis represents sample names and the group classifications. The relative content of differential metabolites across different years is clearly visible. Among these, 19 metabolites, mainly lipids and lipid-like molecules, show a decreasing trend over time, possibly due to microbial degradation. Many differential metabolites, such as isoleucyl-glutamine, pyroglutamyl-valine, and fumaric acid, show minimal differences in abundance between 20-year and 30-year samples. On the other hand, (R)-1-O-[b-D-glucopyranosyl-(1- > 6)-β-D-glucopyranoside]-1,3-octanediol and CDP-DG have the highest relative abundance in the 10-year samples. Cinnamyl B and alpha-linolenoyl ethanolamide show the highest relative abundance in 5-year samples, while their relative abundance remains nearly the same in other years. Vitamin A2 and N-lauroyl histidine have the highest content in 30-year samples. It can be found that the relative content of most metabolites is the highest in older years. 19 compounds (Table 1) can be predicted as characteristic metabolites of Huangjiu with different aging periods.

**3-year-aged Huangjiu** L-Prolinamide, N-docosahexaenoyl Methionine, Artemoin A, Leu-Arg-Asn-Arg, and 3-keto Fusidic acid can be used as characteristic metabolites of 3-year-aged Huangjiu. L-Prolinamide is a derivative of proline, an amino acid that contributes to the umami taste and smooth mouthfeel of Huangjiu. It is likely generated during the aging process through enzymatic or chemical transformations [33]. N-docosahexaenoyl Methionine is a lipid-amino acid that may act as an indicator of lipid oxidation and amino acid metabolism during aging. Artemoin A is a potential bioactive compound that may contribute to the antioxidant properties of Huangjiu [34]. Leu-Arg-Asn-Arg may enhance the savory taste and nutritional value of Huangjiu. Reflects protein degradation and peptide formation during aging. 3-keto Fusidic acid may have antimicrobial properties [35], and could indicate microbial activity during fermentation and aging.

**5-year-aged Huangjiu** Cincassiol B, Alpha-linolenoyl ethanolamide, and Leucyl-Lysine have the highest relative contents in five-year-aged Huangjiu. Cincassiol B is often associated with plant-based materials used in Huangjiu production. May contribute to the herbal or woody aroma of 5-year-aged Huangjiu [36]. Leucyl-Lysine is formed during protein degradation and fermentation, and could contribute to the umami taste and savory flavor of Huangjiu. Alpha-linolenoyl ethanolamide may play a role in mediating the signaling and biological functions of omega-3 fatty acids.

**10-year-aged Huangjiu** CDP-DG, (R)-1-O-[b-D-Glucopyranosyl-(1- > 6)-b-D-glucopyranoside]-1,3-octanediol have the highest relative contents in 10-year-aged Huangjiu. CDP-DG, as a derivative of glycerophospholipids, indicates significant lipid metabolism and oxidative processes occurring during extended aging periods.

**20-year-aged Huangjiu** Oleamide, Salvinolone, and Ginsenoyne A have the highest relative contents in 20-year-aged Huangjiu. These compounds could have bioactive properties, such as antioxidant or antimicrobial effects [37]. Reflects the influence of raw materials and microbial activity during long-term aging. Oleamide, which originates from oleic acid, is commonly linked to lipid metabolism and oxidative reactions [37]. Salvinolone, presumably originating from plant-based ingredients utilized in the production of Huangjiu, could play a role in imparting herbal or woody notes to 20-year-aged Huangjiu. Ginsenoyne A is often associated with ginseng or other medicinal plants.

**30-year-aged Huangjiu** Cinnamylidene acetone, Osmanthuside A, N-lauroyl histidine, Vitamin A2, Coumarin, and Palmitic amide can be used as 30-year-aged Huangjiu marker metabolites. Cinnamylidene acetone is likely formed through the oxidation or condensation of cinnamyl compounds. It may enhance the spicy, woody, or floral aroma profile of 30-year-aged Huangjiu [38]. Osmanthuside A, a glycoside compound, is presumably derived from osmanthus flowers or other botanical ingredients used in Huangjiu production, potentially enriching the floral aroma and sweetness of the wine [39]. N-lauroyl histidine may contribute to the umami taste and smooth mouthfeel of Huangjiu. Vitamin A2, likely originating from raw materials or microbial metabolic processes, could enhance the nutritional profile of Huangjiu. Coumarin, known for its sweet, vanilla-like scent, could contribute to the sweet flavor profile of Huangjiu [40,41].

## 4. Conclusions

This study systematically characterizes metabolite dynamics in Huangjiu across aging periods (3–30 years) using non-targeted metabolomics and high-resolution mass spectrometry. PCA and OPLS-DA revealed significant metabolic differences between aging groups, with 147 differential metabolites identified. Nineteen key metabolites were proposed as aging-specific markers, including lipids, amino acids, and bioactive compounds. These findings provide critical insights into Huangjiu’s flavor evolution and quality control, supporting future research on its health benefits and industrial standardization. Further studies should focus on functional validation and mechanistic exploration of these metabolites in human physiology.

## Figures and Tables

**Table 1:** Characteristic metabolites in different years.

Aging Period	Significant Metabolites
3-year	L-Prolinamide N-docosahexaenoyl Methionine Artemoin A Leu-Arg-Asn-Arg 3-keto Fusidic acid
5-year	Cincassiol B Alpha-linolenoyl ethanolamide Leucyl-Lysine
10-year	CDP-DG (R)-1-O-[b-D-Glucopyranosyl-(1- > 6)-b-D-glucopyranoside]-1,3-octanediol
20-year	Oleamide Salvinolone Ginsenoyne A
30-year	Cinnamylideneacetone Osmanthuside A N-lauroyl histidine Vitamin A2 Coumarin Palmitic amide

## Data Availability

Data are contained within the article.

## Supplementary Materials

The following supporting information can be downloaded at: https://www.mdpi.com/article/10.3390/metabo15050298/s1, Figure S1: The OPLS-DA distribution graph; Figure S2: The OPLS-DA permutation test graph; Figure S3: The base peak ion chromatograms (BPIs) of different QC samples. Table S1: Presents the metabolites that exhibited up-regulation (down-regulation) across all three groups.
