# Supplementary material for: Comparative Analysis of Metabolite Changes in Huangjiu During Different Aging Periods Using HRMS Metabolomics
Source: Metabolites. 2025 Apr 30;15(5):298. doi: 10.3390/metabo15050298 (PMC12112804; doi:10.3390/metabo15050298)
Supplement: Supplementary file 1 [file metabolites-15-00298-s001.zip › metabolites-3571653-supplementary.pdf]

Comparative Analysis of Metabolite Changes in Huangjiu During Different Aging Periods Using HRMS Metabolomics

Yue E, Zhuang Wang and Hongbin Guo

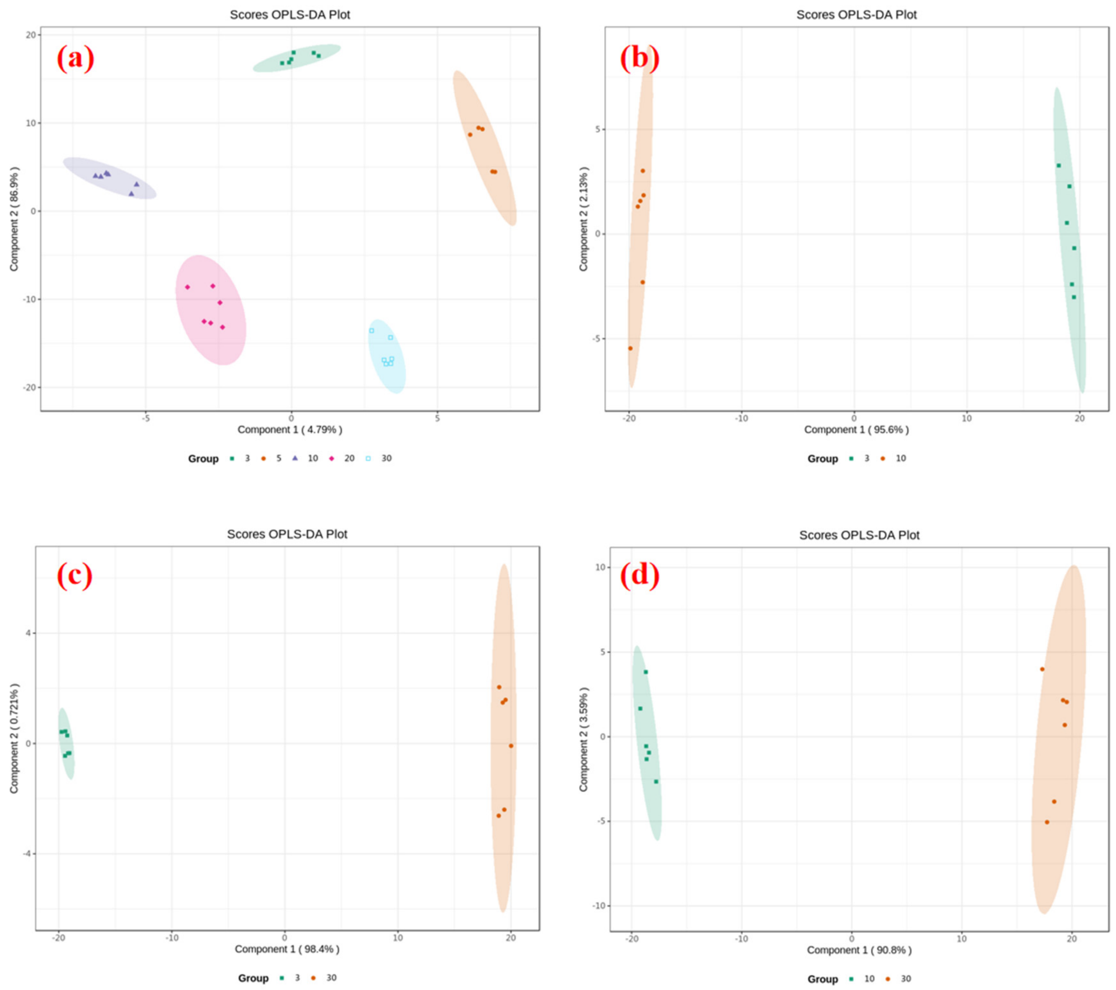

Figure S1. The OPLS-DA distribution graph (a) five different ages, (b) 3-year vs. 10-year, (c) 3-year vs. 30-year, (d) 10-year vs. 30-year.

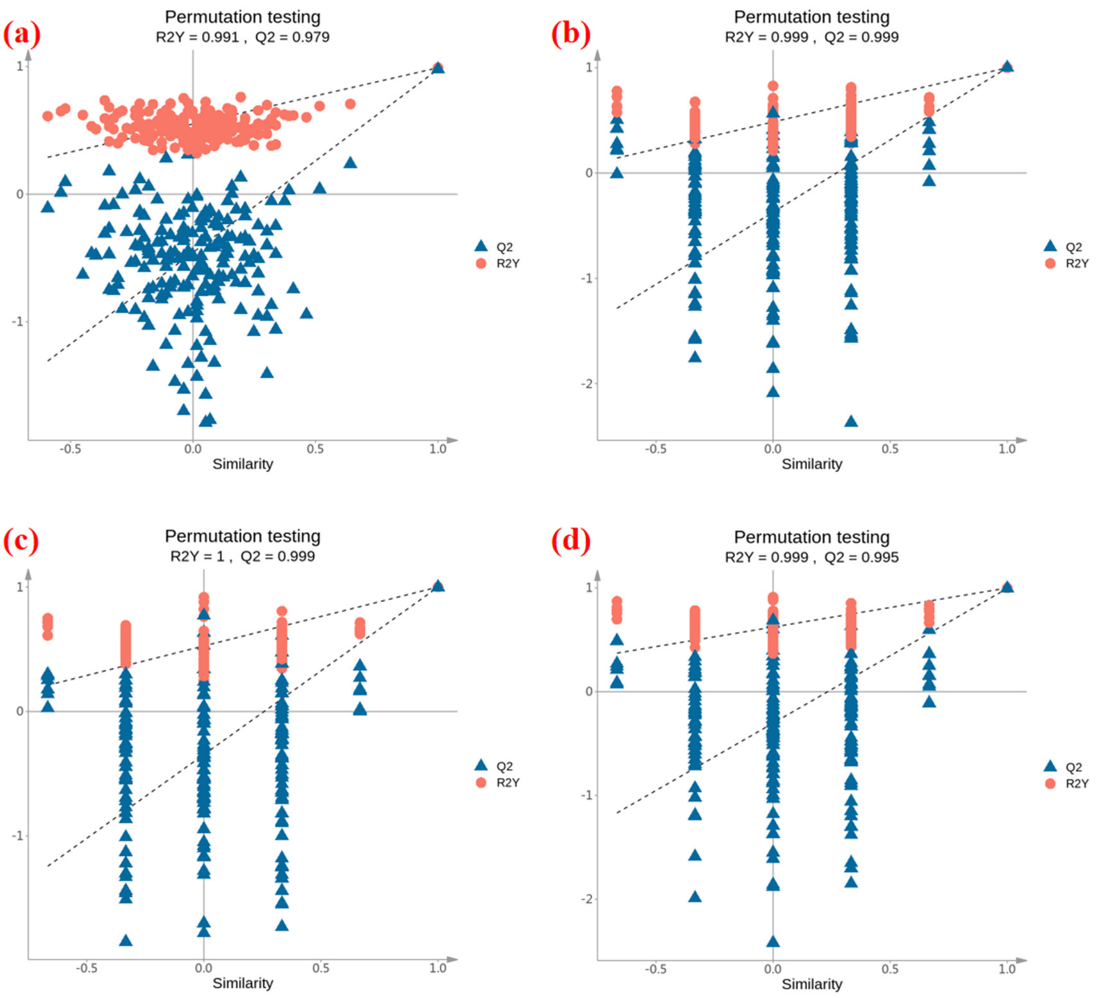

Figure S2. The OPLS-DA permutation test graph (a) five different ages, (b) 3-year vs. 10-year, (c) 3-year vs. 30-year, (d) 10-year vs. 30-year.

(a)

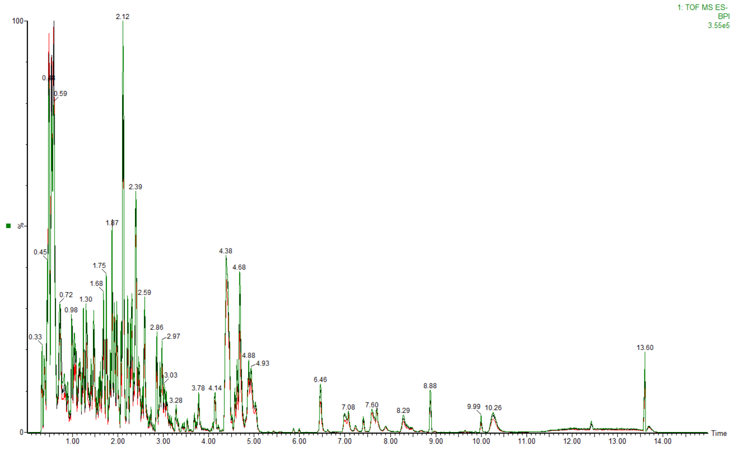

(b)

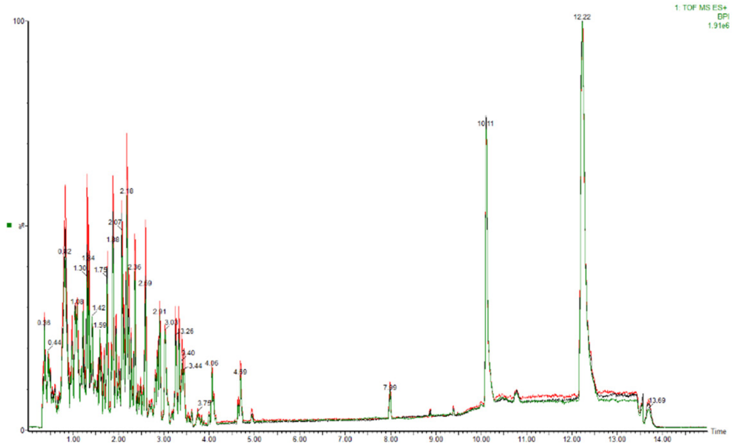

Figure S3. The base peak ion chromatograms (BPIs) of different QC samples. (a) Negative, (b) Positive.

Table S1. Presents the metabolites that exhibited up-regulation (down-regulation) across all three groups.

Trend	Compounds
up	TG(20:0/14:0/18:3(9Z, 12Z, 15Z))
	Cyclopassifloside IV
	PS(24:0/PGF1alpha)
	Leu-Arg-Asn-Arg
	Falcarinolone
down	alpha-Eleostearic acid
	7-Oxomatairesinol
	Artocarpesin
	PC(2:0/22:6(5Z, 8E, 10Z, 13Z, 15E , 19Z)-2OH(7S, 17S))
